# Monkeypox epidemic at the door: should we remain idly by or prepare strongly?

**DOI:** 10.1186/s13568-023-01507-0

**Published:** 2023-01-13

**Authors:** Hayder M. Al-Kuraishy, Ali I. Al-Gareeb, Helal F. Hetta, Athanasios Alexiou, Marios Papadakis, Gaber El-Saber Batiha

**Affiliations:** 1 Department of Clinical Pharmacology and Therapeutic Medicine, College of Medicine, Al-Mustansiriyiah University, Baghdad, Iraq; 2 Department of Medical Microbiology and Immunology, Faculty of Medicine, Assiut University, Assiut, 71515 Egypt; 3 Department of Science and Engineering, Novel Global Community Educational Foundation, Hebersham, NSW 2770 Australia; 4 AFNP Med, 1030 Vienna, Austria; 5 Department of Surgery II, University Hospital Witten-Herdecke, University of Witten-Herdecke, Heusnerstrasse 40, 42283 Wuppertal, Germany; 6 Department of Pharmacology and Therapeutics, Faculty of Veterinary Medicine, Damanhour University, AlBeheira, Damanhûr, 22511 Egypt

**Keywords:** Monkeypox, Pathogenesis, Zoonotic disease

## Abstract

Monkeypox (MPX) is a common zoonotic disease caused by a double-strand DNA MPX virus (MPXV). MPX was considered a sporadic rare disease causing a mild disease with a low capacity to spread among humans. The clinical picture of human MPX highly resembles smallpox, though early lymphadenopathy in human MPX is the distinguishing sign not present in smallpox. The incubation period is 1–3 weeks, and fever, headache, joint pain, myalgia, and nausea for about 3 days. Skin lesions that appear 1–3 days following fever and lymphadenopathy usually appear simultaneously on the face and periphery. By cross-reactivity and protection, the smallpox vaccine produced 85% protection against infection with Orthopoxviruses, including MPX. Antiviral drugs like tecovirimate and brincidofovir could be effective agents against the development of MPX. MPX epidemics are less reported and described as other life-threatening epidemics, leading to an unclear picture of this disease’s pathogenesis, epidemiology, and management. With the recent wide range of MPX outbreaks, immense research is mandatory to revise the importance of MPX pathogenesis and risk for epidemic development worldwide. Therefore, this critical study aimed to review MPX's pathogenesis, epidemiology, and management with possible repurposed drugs.

## Introduction

Monkeypox (MPX) is a common zoonotic disease caused by a double strand DNA MPX virus (MPXV) belonging to the *Orthopoxvirus* genus/*Poxviridae* family.MPXV is highly pathogenic for humans and is recognized as the most important orthopoxvirus infection after the eradication of smallpox in 1980 (Alkhalil et al. 2010; Di Giulio and Eckburg 2004). However, the final and natural host of MPXV is unrecognized, though it infects a wide spectrum of animal and mammalian species. MPX is mainly endemic in the Democratic Republic of Congo (DRC) and some areas of the Ivory Coast (Alkhalil et al. 2010; Di Giulio and Eckburg 2004). Notably, there are two clades of MPXV that differ clinically and epidemiologically. Central African clade (Congo Basin clade) is characterized by a high case fatality rate (CFR) of about 11% with a confirmed person-to-person transmission. Though, West African clade is characterized by low CFR about 1% without person- to-person transmission(Likos et al. 2005).

MPX was considered a sporadic rare disease causing a mild disease with a low capacity to spread among humans. Though, in DRC, it may cause life-threatening complications due to frequent alteration of viral genetic (Yinka-Ogunleye et al. 2018). Albeit, the importance of MPX was less presented previously in the scientific community when it was limited to Central and West Africa. Later on, when MPX was documented outside Africa, a large number of published articles had been increased concerning epidemiology, clinical importance, and risk of epidemics (Sklenovska and Van Ranst 2018).

Moreover, MPX epidemics are less reported and described as other life-threatening epidemics, leading to an unclear picture of the disease's pathogenesis, epidemiology, and management. With the recent wide range of MPX outbreaks, immense research is mandatory to revise the importance of MPX pathogenesis and risk for epidemic development worldwide. Therefore, this mini-review aimed to review MPX's pathogenesis, epidemiology, and management with possible repurposed drugs.

## Epidemiology of MPX

MPX was first recognized in 1958 as a smallpox-like disease in laboratory monkeys in Denmark by Preben von Magnus (Minhaj et al. 2022). The first reported case of human MPX was in 1970, and in 1972 a case of human MPX was identified as a 9-month neonate in DRC (Minhaj et al. 2022). A total of 50 reported cases of human MPX were confirmed between 1970 and 1979, two-thirds of these cases being from DRC (Breman et al. 1980). Meyer et al. reported that by the end of 1986, more than 400 cases of human MPX characterized by 10% CFR were identified in West and Central Africa, which was designed as the first outbreak (Meyer et al. 2002). The second outbreak of human MPX was identified in DRC in a period between 1996 and 1997(Sklenovska and Van Ranst 2018). From 1991 to 1999 a 511 reported cases of human MPX were celebrated in DRC (Sklenovska and Van Ranst 2018).

Notably, MPX is conventionally limited to the tropical rainforest; this pattern was changed in 2003 and 2005 when many cases were recognized in South Sudan and USA that were imported from DRC (Nakazawa et al. 2013). Between 2011 to 2014, about 2000 cases were reported annually in DRC (Sklenovska and Van Ranst 2018). In addition, many interrupted outbreaks were reported from 2014 till the end of 2017. In 2018, an increasing number of suspected cases of human MPX in DRC were reported officially (Sklenovska and Van Ranst 2018). In early May 2022, the first reported case of human MPX was identified in the UK (Nia et al. 2022).

Further, many cases are increased in Europe, Australia, and the USA (Bunge et al. 2022). An updated systematic review illustrated that human MPX endemic in DRC spread and resurged worldwide due to the stopping of smallpox vaccination and increasing rate of person-to-person transmission (Bunge et al. 2022). In addition, an up-to-date human MPX outbreak affects children and young adults compared to the affection of children in early outbreaks (Bunge et al. 2022). These recent observations suggest that human MPX is evolved gradually to become of international relevance.

## Clinical presentation

The clinical picture of human MPX highly resembles smallpox, though early lymphadenopathy in human MPX is the distinguishing sign not present in smallpox. The incubation period is 1–3 weeks, and fever, headache, joint pain, myalgia, and nausea for about 3 days (Minhaj et al. 2022). Skin lesions that appear 1–3 days following fever and lymphadenopathy usually appear at the same time on the face and periphery (Minhaj et al. 2022; Weinstein et al. 2005). Lymphadenopathy is mainly characterized by lymph node enlargement in the neck, groin, and submandibular area. The skin lesions cover the body in severe cases. The skin lesions start as small flat spots that become small bumps (papules) that are filled with clear fluid and then with yellow fluid, sometimes merging to form large lesions. The lesions progress simultaneously, identical to smallpox; after healing, the lesions leave pale marks, which finally become darks (Adler et al. 2022).

Human MPX in severe conditions may lead to various complications, including bronchopneumonia, acute respiratory distress, sepsis, gastrointestinal complications, dehydration, encephalitis, and visual loss due to the involvement of the cornea. Human MPX may be misdiagnosed with chickenpox, smallpox, anthrax and HIV-induced skin lesion (Adler et al. 2022; Adalja and Inglesby 2022). Transmission of MPXV commonly occurs through direct contact and respiratory droplets. Sexual contact with animals could be a possible cause. Skin rashes go in various stages before forming a scab, finally falling out. Two third of patients have skin lesions on the palms and soles (Adalja and Inglesby 2022; World health organization 2022). Diagnosis of human MPX depends on the clinical features and history of contact with animals. Polymerase chain reaction (PCR) of samples from skin lesions is definitive for the final diagnosis. However, blood PCR is not definitive since MPXV no longer persists in the blood (World health organization 2022).

## Pathogenesis of human MPX

**Fig. 1:**
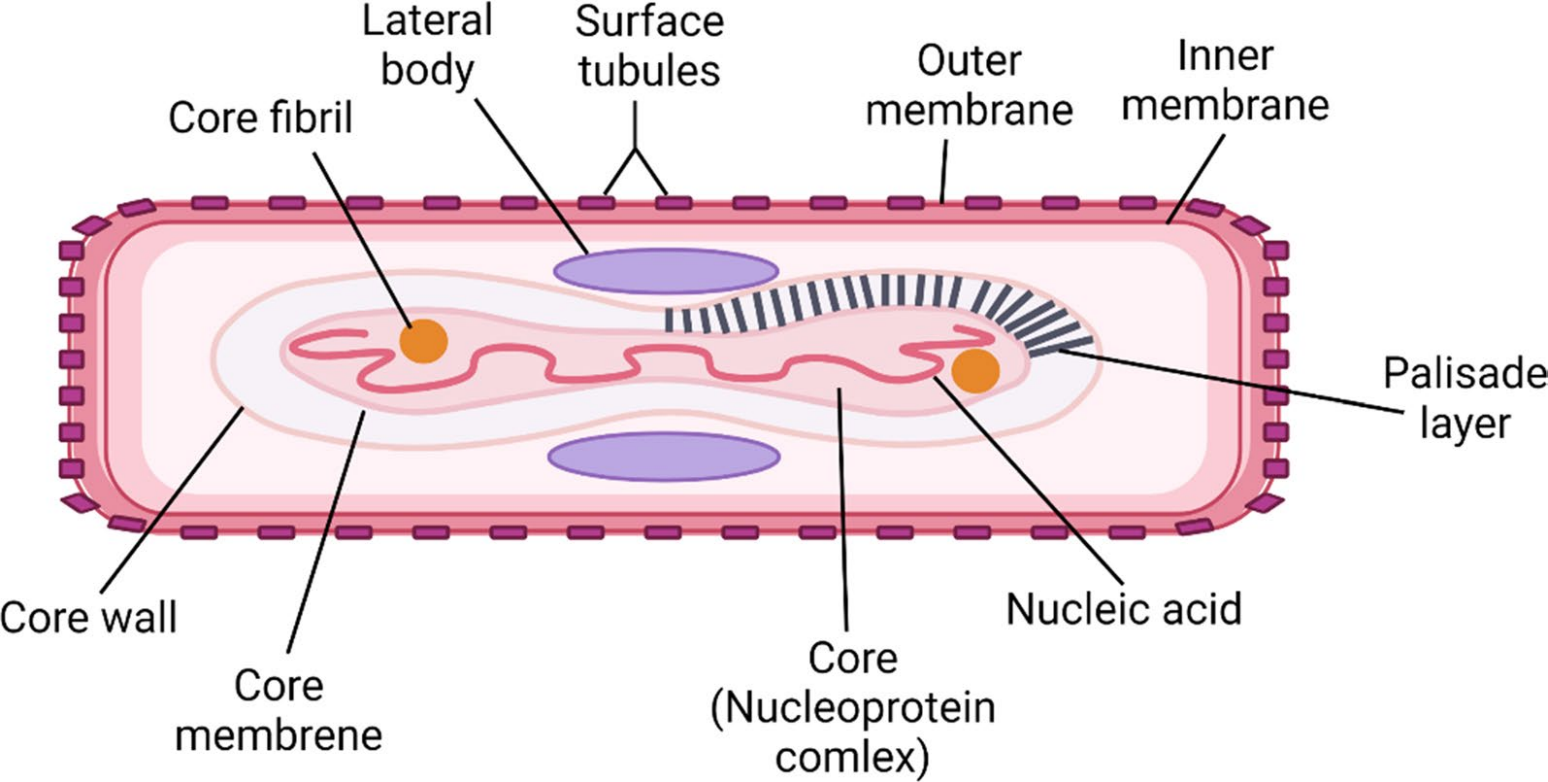
Structure of monkeypox virus

Human MPX is caused by an enveloped dsDNA MPXV which has 250 nm width and 170–250 kb in size of DNA genome. MPXV consists of surface tubules, the outer envelope of the extracellular virion, lateral bodies, the plasid layer, core fibrils, and the outer membrane of intracellular and extracellular virions (Fig. 1).

MPXV enters the mouth, eye, and respiratory tract's mucous membrane (Guarner et al. 2004). Like other *Orthopoxviruses*, entry of MPXV into host cells is achieved by binding glycosaminoglycans (GAGs) on the cell membrane, which mediates viral endocytosis (Kabuga and El Zowalaty 2019). Viral RNA polymerase is expressed within 30 min following infection with this virus. In the cytoplasm, the viral core and genomes are released with subsequent replication of viral proteins by viral DNA polymerase. Viral structural proteins are produced within 48 h post-infection with subsequent assembly into mature virions in the Golgi apparatus. From the Golgi apparatus, the mature virions are transported by microtubules to the plasma membrane and released outside the infected cells to infect other cells similarly (Likos et al. 2005).

There are four types of GAGs, including heparin sulfate, chondroitin/dermatan sulfate, keratin sulfate, and hyaluronic acid (Gandhi and Mancera 2008). Heparin sulfate is present in the mast cell granules and is involved in regulating cell growth and proliferation, viral invasion angiogenesis, and tumor metastasis. Keratin sulfate maintains tissue hydration and regulation of macrophage functions. Other types of GAGs are found in the connective tissues and synovial membranes (Gandhi and Mancera 2008; Sodhi and Panitch 2020) GAGs are complex carbohydrates ubiquitously expressed on the cell surface and extracellular matrix. The interactions between GAGs and microbial pathogens represent a defense line against invasion (Lin et al. 2020). Several pathogens, including MPXV, induce the release of GAGs with the formation of soluble GAGs, which coat the pathogen to escape immune detection (Akhtar and Shukla 2009). Heparin sulfate acts as an entry and attachment site in the respiratory tract for many viruses, such as human meta-pneumonia, respiratory syncytial, and coronaviruses (Akhtar and Shukla 2009). GAGs and heparin sulfate, together with sialic acid, are abundantly expressed in dermal and epidermal cells that facilitate binding and entry of MPXV to the skin (Hughes et al. 2014).

**Fig. 2:**
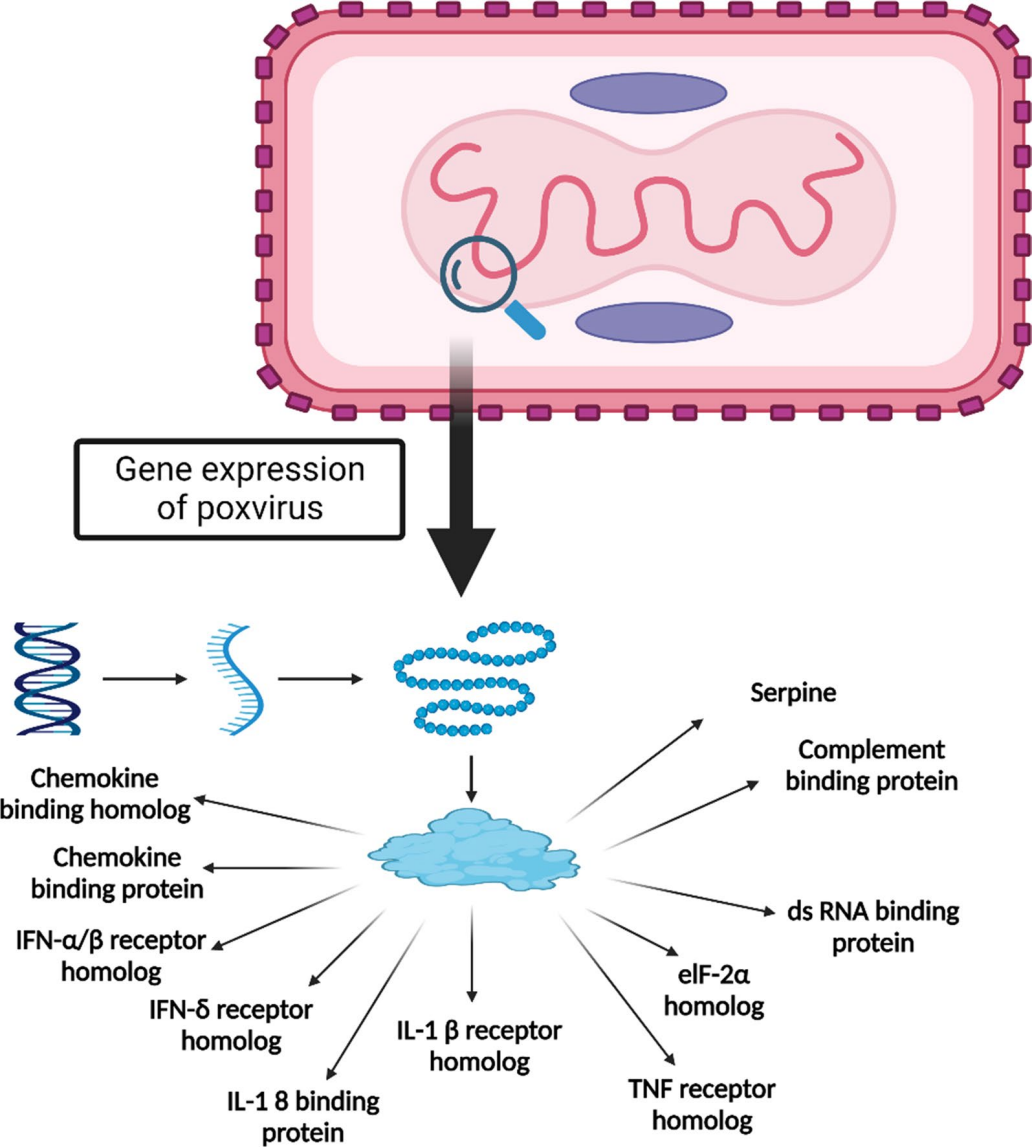
Viroceptors and virokines of poxvirus

In MPXV, like other *Orthopoxviruses* there is noteworthy activation of genes involved in the activation expression of pro-inflammatory cytokines and chemokines like IL-6 and CCL-2, respectively (Bourquain et al. 2013). Besides, mitogen-activated protein kinase (MAPK) and extracellular signal-regulated kinase (ERK) are induced by MPXV, which promote viral entry, viral replication and increased expression of viral proteins needed for viral replication (DuShane and Maginnis 2019). Moreover, heat shock protein 1 (HSP-1) is necessary for replicating MPXV, so it is highly induced during infection with MPXV (Filone et al. 2014). Of note, MPXV induces activation of nuclear factor kappa B (NF-κB) via suppression of signal transducer and activator of transcription (STAT), which has antiviral effects (Filone et al. 2014). In addition, MPXV activates natural killer cells to release interferon-gamma (INF-γ) and tumor necrosis factor-alpha (TNF-α), which induce T-helper immune response type 1 (Th1) (Townsend et al. 2013). Overall, due to the selective tropism of lymphoid tissues, MPXV can induce lymphopenia and lymphadenopathy (Townsend et al. 2013). Inhibition of CD4 and CD8 and retaining of major histocompatibility complex 1 (MHC1) could be the possible mechanism for immune evasion of MPXV (Hammarlund et al. 2008). MPXV can evade the immune response by releasing receptors and virokines identical to the host cytokines. Viroceptors and virokines interfere with the intracellular signaling pathways of infected cells (Fig. 2) (Hammarlund et al. 2008).

These observations may explain the immunosuppressive effect of MPXV, as evidenced by the reduction of T lymphocytes in MPX (Hammarlund et al. 2008). Therefore, unlike other viral infections, the immune response is complex and needs further attention.

## Management of MPX

### Antiviral agents

Tecovirimate is approved in the USA and the European Union for treating various types of poxviruses, including MPXV (Adler et al. 2022). Tecovirimate inhibits virus release from infected cells by inhibiting envelop proteins required for virion release. This antiviral drug was first used in 2018 for vaccinia. According to many clinical trials, it has proven to be more effective against MPXV. This drug is safe and well-tolerable (Grosenbach et al. 2018). As well brincidofovir is also recommended as a first-line antiviral drug together with supportive treatment in the management of MPX (Adler et al. 2022). Brincidofovir is a prodrug of cidofovir, conjugated with lipids designed to release cidofovir within the cells allowing higher intracellular concentration. Brincidofovir inhibits the DNA polymerase of MPXV and the production of extracellular viruses (Ho et al. 2022). It was approved in the USA in 2021 for treating poxviruses like adenoviruses, Ebola, and cytomegalovirus (Ho et al. 2022). The most common adverse effects of brincidofovir are nausea, vomiting and abdominal pain (Ho et al. 2022). Brincidofovir has greater efficacy than cidofovir by about 25 folds (Ho et al. 2022; Hutson et al. 2021).

Other possible antiviral drugs for treating MPX are tricyclodicarboxylic acid (a derivative of tecovirimat), ribavirin, tiazofurin, protease inhibitors and hydrolase inhibitors could be effective in the management of MPX (Hutson et al. 2021; Lal et al. 2021).

### Vaccination

It has been shown that the smallpox vaccine produced 85% protection against infection with *Orthopoxviruses,* including MPX, by cross-reactivity and protection (Yoshikawa 2021). Therefore, vaccinated individuals with the smallpox vaccine are more protected against the development of MPX. The smallpox vaccine was recommended in the 2003 MPX outbreak in the USA to reduce the risk of disease transmission (Choi et al. 2021). However, propaganda against the smallpox vaccine was high in 2003 due to the development of uncertain adverse effects like hiccup, mainly in immune-compromised patients (Kaynarcalidan et al. 2021). Later, IVAMUNE and ACAM2000 smallpox vaccines were developed and were shown to be not contraindicated in immune-compromised patients (Nalca and Zumbrun 2010). Till now, a specific vaccine for MPX is not developing, decussating findings of specific antiviral drugs or drugs with a potential effect on the development of MPX from repurposed ones.

We learned many lessons from the Covid-19 pandemic, like the rapid involvement of repurposed FDA-approved drugs like ivermectin and doxycycline in managing Covid-19 (Al-Kuraishy et al. 2020; Al-Kuraishy et al. 2021a, b; Al-Kuraishy et al. 2021a, b). In addition, lymphopenia and abnormal immune response in Covid-19 may increase the risk for the development of MPX. In this regard, many safe drugs should be tested in silico and experimental studies when preparing preclinical studies.

In conclusion, Monkeypox (MPX) is a common zoonotic disease caused by a double-strand DNA MPXV. MPX was considered a sporadic rare disease causing a mild disease with a low capacity to spread among humans. With the recent wide range of MPX outbreaks, immense research is mandatory to revise the importance of MPX pathogenesis and risk for epidemic development worldwide. By cross-reactivity and protection, the smallpox vaccine produced 85% protection against infection with Orthopoxviruses, including MPX. Antiviral drugs like tecovirimate and brincidofovir could be effective agents against the development of MPX. Till now, a specific vaccine for MPX is not developing, a decussating finding of specific antiviral drugs or drugs with a potential effect on the development of MPX from repurposed one.

## Data Availability

All generated data are included in this manuscript.
